# Disturbed microcirculation in the hands of patients with systemic sclerosis detected by fluorescence optical imaging: a pilot study

**DOI:** 10.1186/s13075-017-1300-6

**Published:** 2017-05-08

**Authors:** Stefanie Friedrich, Susanne Lüders, Stephanie Gabriele Werner, Anne-Marie Glimm, Gerd-Rüdiger Burmester, Gabriela Riemekasten, Marina Backhaus, Sarah Ohrndorf

**Affiliations:** 1 Department of Rheumatology and Clinical Immunology, Charité University Hospital, Berlin, Germany; 2 Department of Rheumatology, University of Schleswig-Holstein, Lübeck, Germany; 3 Department of Internal Medicine - Rheumatology and Clinical Immunology, Park-Klinik, Weißensee, Berlin Germany; 4 Rheumatologie, Immunologie und Osteologie mit, Schwerpunkt für Rheumatologie, Klinische Immunologie und Osteologie am Ev. Krankenhaus Düsseldorf, Düsseldorf, Germany

**Keywords:** Fluorescence optical imaging, Systemic sclerosis, Raynaud’s phenomenon, Disturbed microcirculation, Digital ulcers, Nailfold capillaroscopy

## Abstract

**Background:**

Utilising fluorescence optical imaging (FOI), the distribution of an intravenously applied colouring agent indocyanine green (ICG) can be analysed with the potential to identify malperfusion by little to no tissue enhancement. Systemic sclerosis (SSc) is characterised by the presence of digital ulcers reflecting progressive vasculopathy.

The objective was to investigate the potential of FOI in the detection of disturbed microcirculation in the hands and fingers of patients with SSc and to link FOI findings to clinical signs of ischemia such as digital ulcers and pitting scars.

**Methods:**

In this cross-sectional study, 63 patients with SSc and 26 healthy subjects were examined. FOI was performed in all 89 individuals and compared to clinical data and capillaroscopic findings assembled for the SSc cohort.

**Results:**

Healthy subjects showed initial ICG signals in their fingertips in 93.6%, SSc patients in 78.5% (limited SSc) and 43.2% (diffuse SSc). Moreover, in SSc patients, FOI findings were significantly associated with a late capillaroscopic pattern, disseminated SSc features, a diffuse SSc subtype, and the presence of digital ulcers or pitting scars. Intra- and inter-reader reliability for FOI amounted to κ = 0.786 and κ = 0.834, respectively.

**Conclusions:**

FOI is able to detect areas of reduced microcirculation in patients with SSc with high association to capillaroscopic findings. The results pave the way for future FOI investigations into its role in the prediction of complications due to an impaired acral perfusion.

## Significance and innovations

- FOI shows areas of diminished acral perfusion in patients with systemic sclerosis
- FOI presents a significant association between diminished perfusion and clinical signs of ischemia in SSc
- Diminished perfusion in FOI is significantly linked to capillaroscopic patterns in SSc
- The prevention of digital ulcers in SSc using FOI should be investigated

## Background

Indocyanine green (ICG)-enhanced fluorescence optical imaging (FOI) is a novel, fast and quantitative imaging method that is already used in the diagnostics of inflammatory arthritis with good agreement to other imaging modalities (magnetic resonance imaging [MRI], ultrasound [US]) and good reliability [1–3]. Light of the (near)-infrared spectrum enters the tissue of the hands, where it stimulates the fluorophore ICG. Its fluorescence is then detected by a charge-coupled detector (CCD). A locally increased microcirculation, e.g. in inflamed joints, leads to strong signal enhancement, due to higher ICG concentration.

ICG fluorescence video angiography has also been shown to help in the detection of compromised tissue perfusion as a perioperative tool in microsurgery [4].

ICG is well tolerated and side effects (i.e. anaphylactic symptoms) occur rarely (1:42,000) [5]. Shortly after its administration, ICG is hepatically metabolised and hepatobiliarily excreted. Already since the 1970s, ICG has safely been used in ophthalmologic angiography and even proved its value in the evaluation of skin perfusion in plastic surgery [6].

Pfeil et al. introduced FOI in SSc patients. Compared to healthy subjects, areas with strong signal enhancement could be observed and were interpreted as tissue inflammation. A normalisation occurred after prostacyclin treatment [7]. However, these investigations focused on areas of increased perfusion.

Patients with systemic sclerosis (SSc) typically suffer from Raynaud’s phenomenon (RP) and progressive obliterative vasculopathy. Both lead to a decreasing or even completely ceasing circulation in distal body parts, i.e. fingertips or the nose. Almost one third of SSc patients consequently develop at least one digital ulcer per year, [8] which usually causes pain, loss of function, local and systemic infection and reduced quality of life.

So far, several predictive factors for the development of digital ulcers (DU) have been described, i.e. diffuse subtype of SSc, late pattern of capillaroscopy, history of DU or markers for endothelial dysfunction [9–12]. Various imaging methods have been suggested to measure vasculopathy in patients with systemic sclerosis and to link its extent to the existence of current or future digital ulcers. Endothelial function assessed by multisite photoplethysmography (PPG), cardiovascular assessments and the peripheral blood perfusion assessed by laser speckle contrast analysis (LASCA) were lower in SSc patients with digital ulcers than in patients without [13, 14]. Laser Doppler perfusion imaging is able to detect the therapeutic effects of Bosentan, but could not differentiate between patients with and without digital ulcers [15]. The best prospective data exist for nailfold capillaroscopy (NC): the capillaroscopic skin ulcer risk index (CSURI) has a high specificity (81.4%) and sensitivity (92.98%) for new or persistent digital ulcers after 3 months (less so in clinical settings without limitations as to vasoactive treatment), but can only be applied in patients with megacapillaries [16, 17]. A pilot study of Smith et al. introduced the assessment of capillaries in eight specific regions in regard to present and future digital ulcers [18]. The videoCAPillaroscopy (CAP) multicentre study found the number of capillaries in the middle finger of the dominant hand among other capillaroscopic covariables to be a predictive factor for the development of digital ulcers [19]. Other studies focused on capillaroscopic patterns (i.e. early, active and late) and found associations to the presence as well as the development of digital ulcers [11, 12, 20–24]. While these studies have not provided concrete values of specificity and sensitivity, we calculated levels of around 36% and 89% respectively using their provided data [11, 12, 20, 23, 24].

To our knowledge, there has not yet been any investigation into FOI’s potential to detect areas with disturbed or decreased microcirculation in SSc patients. We therefore investigated whether diminished acral perfusion – as found in patients with systemic sclerosis – was assessable in FOI, i.e. via areas of reduced enhancement. This is contradictory to former research using fluorescence optical imaging as the main focus so far has been placed on increased signal intensity typical for inflammation. FOI findings in SSc patients were compared to those in healthy individuals and associated with their SSc subtype, capillaroscopic patterns, 2013 ACR/EULAR criteria, clinical findings as well as pitting scars (PS) and DU at baseline as defined by Medsger et al. [25].

## Methods

### Subjects’ characteristics

Between 2013 and 2015, 89 individuals participated in this study forming two cohorts: the SSc group and the healthy cohort. We consecutively included in- and outpatients fulfilling the 2013 ACR/EULAR criteria for systemic sclerosis. Healthy subjects were defined as persons without any known diseases, e.g. vascular diseases. For subjects’ characteristics see Table 1. Subjects with current DU/PS were summarised in group 1, subjects without DU/PS in group 2. Exclusion criteria were progressive renal dysfunction (glomerular filtration rate [GFR] ≤15 ml/min), latent and manifest hyperthyroidism, allergies to iodine or contrast agents, pregnancy and being underage. 2013 ACR/EULAR classification guidelines were applied with seven different criteria each scoring 2, 3, 4 or 9 points (such as present DU/PS, RP or skin induration) [26]. A maximum of 30 points could be obtained; ≥9 points confirmed the classification as SSc. We defined the range of scores between 9 and 30 points as a marker for dissemination of SSc features: a higher individual ACR/EULAR score meant the presence of more SSc characteristics. Limited, limited cutaneous and diffuse cutaneous SSc (lSSc, lcSSc and dcSSc) were distinguished according to LeRoy et al. [27].

**Table 1:** Characteristics of subjects

Subjects’ characteristics	Systemic sclerosis (SSc)	Limited (cutaneous) SSc	Diffuse cutaneous SSc	Healthy cohort
	n = 63	n = 40	n = 23	n = 26
Female: n (%)	52 (82.5%)	35 (87.5%)	17 (73.9%)	19 (73.1%)
Mean age in years (±SD)	56.4 (±14.4)	56.4 (±14.9)	56.6 (±13.4)	28.8 (±9.6)
Mean disease duration (in years ± SD) since				
First Raynaud’s	13.3 (±12.8)	14.6 (±12.3)	11.1 (±13.6)	Not present
First non-Raynaud’s symptoms	9.8 (±9.4)	9.7 (±9.6)	9.8 (±9.3)	Not present
Patients with Raynaud’s in the past week				
Number of patients (%)	57 (90.4%)	36 (90.0%)	21 (91.3%)	Not present
Mean number of episodes (±SD)	15.5 (±22.4)	17.5 (±27.3)	11.9 (±8.8)	
Mean impact on day-to-day life using VAS 0-100 (±SD)	36.3 (±29.6)	37.0 (±29.7)	35.1 (±30.1)	
Digital ulcers/pitting scars, n (%)	37 (58.7%)	18 (45.0%)	19 (82.6%)	Not present
Skin involvement				
Mean mRSS ± SD	9.0 (±8.2)	4.6 (±3.5)	16.5 (±8.6)	Not present
Organ involvement, n (%)				
Lung	23 (36.5%)	9 (22.5%)	14 (60.9%)	Not present
Gastrointestinal	42 (66.7%)	25 (62.5%)	17 (73.9%)	
Heart	10 (15.9%)	3 (7.5%)	7 (30.4%)	
Capillaroscopy pattern, n (%) [21]				
Early	11 (17.5%)	9 (22.5%)	2 (8.7%)	
Active	25 (39.7%)	18 (45.0%)	7 (30.4%)	
Late	24 (38.1%)	11 (27.5%)	13 (56.5%)	
Non-SSc	1 (1.6%)	1 (2.5%)	0	
Not done	2 (3.2%)	1 (2.5%)	1 (4.3%)	26 (100%)
Current medication, n (%)				
Iloprost	38 (60.3%)	27 (67.5%)	11 (47.8%)	0
Oral vasodilating drugs^a^	42 (66.7%)	25 (62.5%)	17 (73.9%)	0

One patient with limited systemic sclerosis (SSc sine scleroderma) was included in the limited cutaneous SSc subgroup

*VAS* visual analogue scale, *mRSS* modified Rodnan skin score

^a^Calcium channel blockers, AT1 receptor antagonists, ACE inhibitors, endothelin receptor antagonists, PDE-5 inhibitors; alpha receptor antagonists

Organ involvement was evaluated as follows: manifestations of the lung were either pulmonary hypertension or interstitial lung disease, determined by right heart catheterisation or echocardiogram and high-resolution computed tomography or lung function test, respectively. Gastrointestinal involvement was defined as dysphagia and/or radiographic dysmotility and chronic diarrhoea. Cardiac involvement comprised arrhythmias, congestive cardiac failure, pericardial effusion, and conduction defects not attributable to other cardiac conditions.

### Fluorescence optical imaging

The FOI method Xiralite X4 (mivenion GmbH, Berlin, Germany) measures the fluorescence of the fluorophore indocyanine green (ICG, 5% iodine) using light-emitting diodes and a charge-coupled detector (CCD, a high-resolution camera). Light with defined wavelengths (ca. λ = 740 nm) in the (near)-infrared spectrum is emitted and sent through the observed tissue stimulating the fluorophore (if present). The fluorophore then emits fluorescence of different wavelengths (max. λ = 832 nm) whose photons are detected by CCD. Refracted and reflected light is filtered out, so that the resulting image represents a true depiction of ICG distribution in the tissue [1, 3].

Thus, the method permits a visualisation of different ICG quantities in the tissue of both hands, e.g. higher quantities of the agent can be found in inflamed tissue due to capillary leakage as well as an increase in blood flow and vessel density on a microvascular level [28]. Low ICG quantity – and therefore low fluorescence signal – results from poor tissue perfusion.

All participants in this study received FOI examination following the Xiralite system guidelines (ICG 0.1 mg/kg BW intravenously, 6 minutes’ duration). Subjects had to wait in an environment of 20–23 °C for 20 minutes in order to diminish the risk of an acute Raynaud’s episode.

A peripheral venous catheter, the colouring agent (0.1 mg/kg BW) as well as a syringe with 10 ml NaCl 0.9% solution were prepared. The subject’s hands were then placed on a mould underneath the camera with a curtain lying on the lower arms in order to diminish disturbance by exterior light. The examiner (e.g. a medical assistant) checked for possible disturbances (e.g. exterior light, spilled ICG) watching the XiraView® preview, before starting the examination. FOI began with ten (reference) images being taken before ICG administration. Immediately afterwards, 10 ml NaCl 0.9% solution was applied to introduce the complete amount of ICG into the blood stream, where ICG binds to 98% onto plasma proteins. Indocyanine green has a half-life of 3.4 minutes (±0.7) and is hepatically eliminated with a mean decay of 18.5% per minute (±2.1); 20 minutes after applications, studies have shown a mere trace (3.8% ± 1.0 of the original dose) to still be found in the body [28]. The infrared camera continued taking one picture per second for the next 350 seconds, resulting in a total examination time of 6 minutes. With ICG’s half-life span and rate of decay one can expect the fluorophore to have passed through the bloodstream and accumulated in the observed tissue in this timespan.

### FOI analysis

XiraView® was used to conduct the analysis, especially its 360-second-long clip mode, which depicts ICG distribution in the hands after administration. The *PrimaVistaMode*, a summation image of the examination, was used for visualisation in this work’s figures.

The number of photons emitted from the fluorophore is recorded in every pixel. After the examination, these are proportioned to the reference images (no ICG) and the pixel with the highest photon emission in the entire examination. The percentage of the actual emission in the pixel compared to the maximum emission is used by the software to create a colour scale for optimal visualisation which we used for our analysis: white and red signals indicate a strong enhancement (= strong ICG accumulation in the tissue defined as ≥65% of the individual maximum quantity), yellow and green signals indicate a medium enhancement, light blue signals show lower ICG accumulation in the tissue, dark blue signals show very little ICG accumulation and no ICG accumulation at all is coded in black. A dark blue or black signal was considered as an insufficient enhancement due to low ICG accumulation (<27% of the individual maximum quantity) in the region (to see the defined regions please compare Fig. 4) observed. The cutoffs of >65% and <27% for strong and insufficient signal respectively were provided by the software as 65% defines the mark between the red and yellow signal and 27% the mark between the light and dark blue signal.

**Fig. 1:**
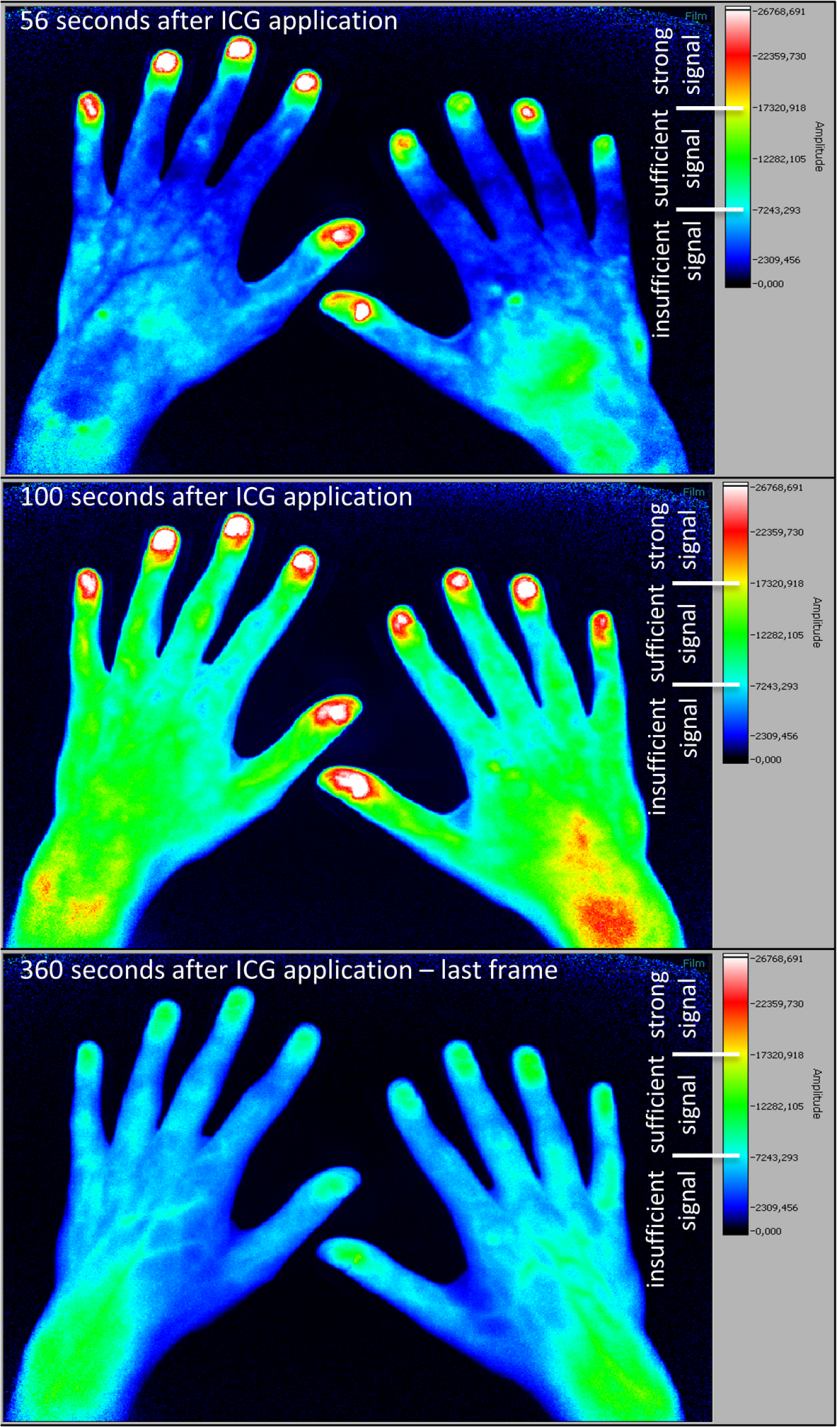
Fluorescence optical imaging (FOI) staining in a healthy subject. *Upper image*: initial enhancement (IE), defined as the first strong signal of indocyanine green (*ICG*), in digit II of the right hand; IE in the left hand has already taken place. *Middle image*: area of maximum distal distribution (MDD) is the fingertip (region 0) in every finger as this is the most distal region with a sufficient ICG signal. *Lower image*: last image (no. 360) of the examination; most of the ICG has already disappeared

**Fig. 2:**
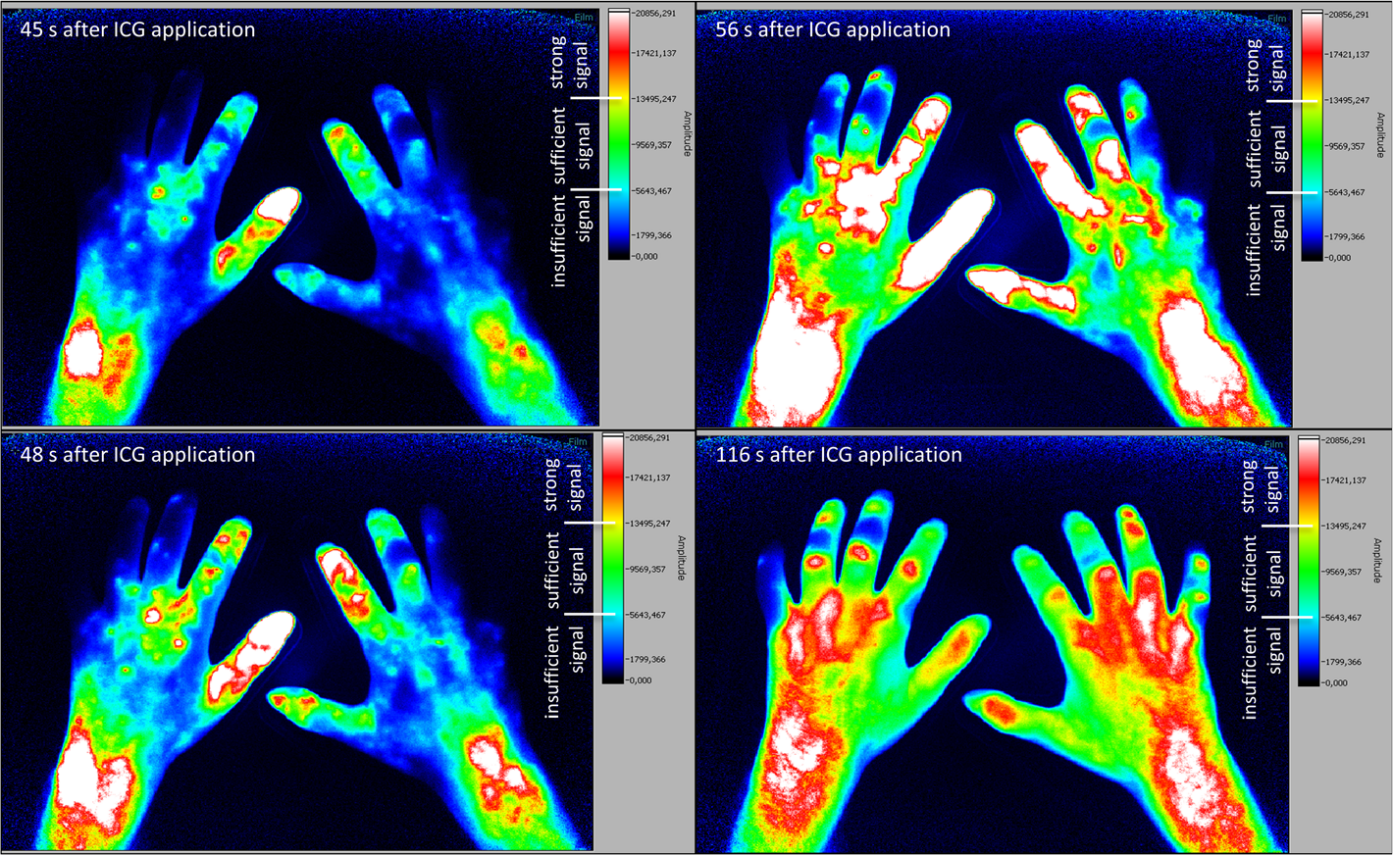
Fluorescence optical imaging (FOI) staining in a patient with diffuse cutaneous systemic sclerosis. *Upper left image*: strong initial enhancement (IE) in digit II of the right hand just below the fingertip (region 1). *Lower left image*: IE in the digit III of the left hand near the metacarpophalangeal (MCP) joint (region 6). *Upper right image*: maximum distal distribution (MDD) in the fingertip (region 0) of digit II of the right hand. *Lower right image*: MDD in digit V of both hands with the most distal sufficient signal in region 5 of the left digit V and region 1 of the right hand’s digit V. *ICG* indocyanine green

**Fig. 3:**
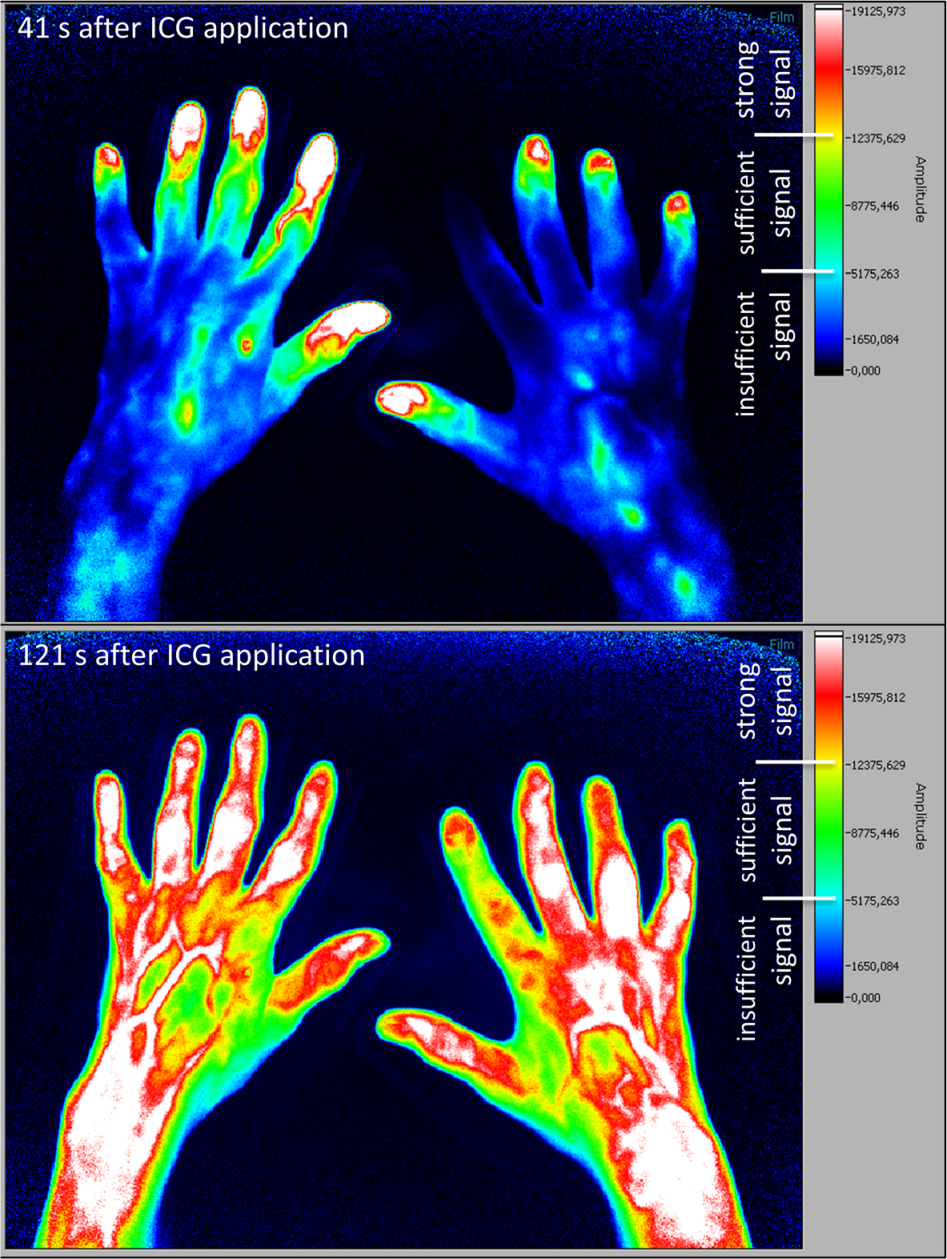
Fluorescence optical imaging (FOI) staining in a patient with lcSSc, without clinically apparent Raynaud’s episode. *Upper image*: strong initial enhancement (IE) in digits I, III, IV and V of the right hand; the index finger has not yet accumulated indocyanine green (*ICG*). *Lower image*: maximum distal distribution (MDD) in the fingertip of the right hand’s index finger

Each finger of every subject was separately inspected (see Figs. 1, 2 and 3)

We evaluated three things in particular:

1. The region of initial enhancement (IE), which was defined as a finger’s first region presenting a strong ICG accumulation (≥65% of the individual maximum quantity; coded as a white or red signal). Ideally, this region would be the fingertip (region 0, compare Fig. 4), designating an unperturbed arterial blood flow. A strong signal below the fingertip would depict the last area with (supposedly) unperturbed circulation.

**Fig. 4:**
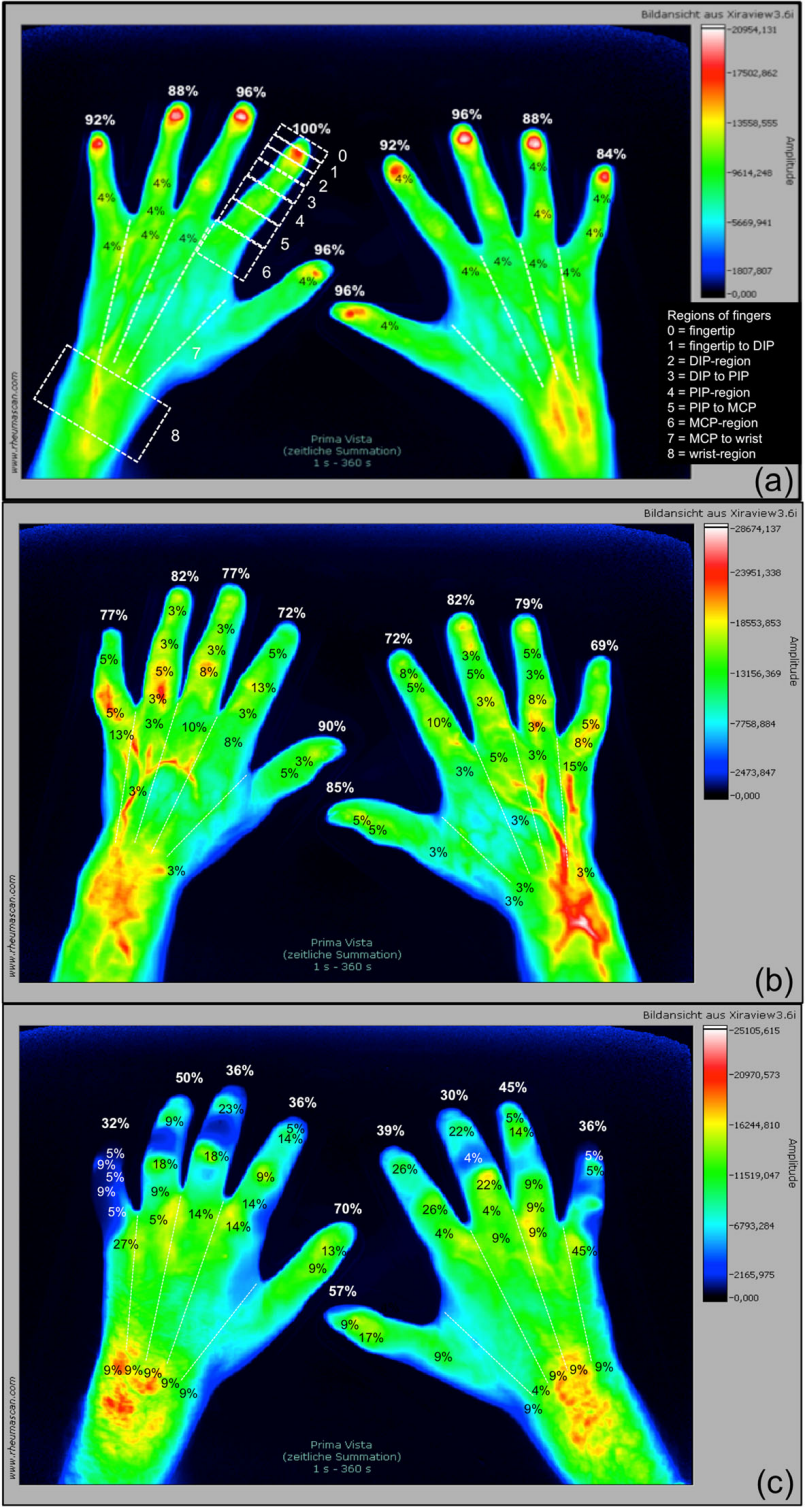
Regions of initial enhancement in healthy subjects and systemic sclerosis patients. *PrimaVistaMode* (summation image) of (**a**) a healthy subject and exemplary depiction of the nine regions in digit II, left hand with finger-wise percentage of initial enhancement of indocyanine green (ICG) of the healthy cohort: proper distribution of the colouring agent with 84–100% (mean: 93.1%) of first ICG signals in region 0 (fingertip). *PrimaVistaMode* of a patient with (**b**) limited (cutaneous) systemic sclerosis (lcSSc) and (**c**) diffuse cutaneous systemic sclerosis (dcSSc) and depiction of the finger-wise percentage of strong initial ICG enhancement per region (0–8). LcSSc cohort with 69–90% (mean: 78.5%) and dcSSc cohort with 30–70% (mean: 43.2%) of initial ICG signals in region 0. *DIP* distal interphalangeal joint, *MCP* metacarpophalangeal joint, *PIP* proximal interphalangeal joint
2. The region of maximum distal distribution (MDD), which was defined as the most distal region that shows sufficient ICG enhancement (≥27% of the individual maximum quantity; coded as a white, red, yellow, green or light blue signal) during the course of the examination. Ideally, this region would also be the fingertip (region 0). Signals below would mean an insufficient provision of blood to more distal regions due to angiopathy, occlusion etc.
3. Complete disruption: a complete disruption of acral microcirculation was defined as an insufficient ICG accumulation (i.e. <27% of maximum signal intensity) in the fingertip of at least one of the subject’s finger during the entire examination. That implies that the region of MDD is more proximal than the fingertip (i.e. regions 1 to 8, compare Fig. 4).

We assigned eight regions (region 0 to region 7) to each finger (thumbs: five regions) starting with the fingertip (region 0) and ending in the metacarpal region. The wrist was considered a common region 8 (see Fig. 4).

Furthermore, ICG accumulation patterns for typical changes during IE as described by Granzow et al. [29] were scanned. The examination and analysis as described were performed in a maximum of 15 minutes per subject.

### Nailfold capillaroscopy

**Fig. 5:**
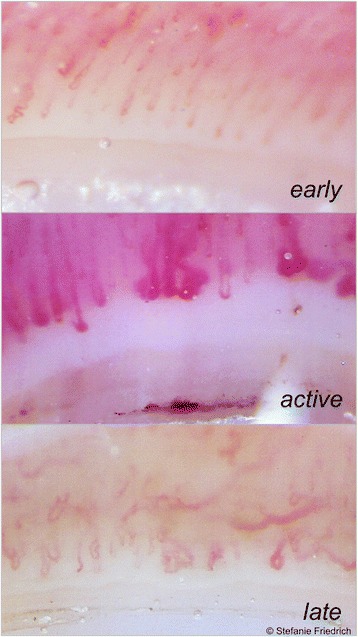
Typical nailfold capillaroscopy patterns in systemic sclerosis patients. Exemplary images of the three patterns of nailfold capillaroscopy as defined by Cutolo et al. [29]: the early pattern (*upper image*) is characterised by a normal capillary density and distribution with few ectasias and haemorrhages. The active pattern (*middle image*) is defined by giant capillaries, many haemorrhages and mild capillary loss and disorganisation. Irregular capillary width, avascular areas due to decreased capillary density as well as ramified or bushy capillaries, but less giant capillaries and haemorrhages constitute the late pattern (*lower image*)

This method was conducted with a USB device (Di-Li 970-O USB hand microscope Di-Li®-Lite; Bresser, Rhede, Germany) and the MicroCapture V2.0 software (to determine the capillary density) following current guidelines. Three distinct capillaroscopic SSc patterns (early, active, late pattern, compare Fig. 5) as well as non-SSc-specific findings were identified as defined by Cutolo et al. [21]. An exact examination via nailfold capillaroscopy (NC) usually took our examiners 40 minutes per subject.

### Statistical analysis

Statistical analysis was conducted by the statistical software SPSS (Version 21.0.0.2; IBM Corp, Armonk, NY, USA). Mann-Whitney *U*, Fisher’s exact and X^2^ tests were performed. A *p* value of ≤0.05 was considered statistically significant.

### Intra- and inter-reader reliability

A trained FOI examiner (SF), who assessed the initial FOI findings directly after the conducted examination, re-evaluated 20 consecutive patients’ findings in January 2016. A second examiner (SL), who was trained by the first reader, separately evaluated the FOI findings of these 20 patients. Both examiners were blinded to the initial results as well as the results of one another. Intra- and inter-reader reliabilities were calculated using Cohen’s kappa test.

## Results

### Healthy subjects present sufficient ICG enhancement in all digital regions

In the healthy cohort, 242 of 260 fingers (93.1%; Fig. 1) showed a strong initial enhancement (IE, ≥65% of maximum signal intensity) in the fingertips (region 0) with a subsequent expansion of ICG to more proximal areas (region 1 to region 8; Fig. 4). The other 18 (6.9%) fingers of the healthy cohort presented a sufficient ICG accumulation (>27% of maximum signal intensity) in the fingertips later on, designing a sufficient maximum distal distribution (MDD) in the entire healthy cohort.

### Pathologic ICG accumulation is related to general presence of digital ulcers and/or pitting scars in SSc patients

We observed a strong (≥65% of maximum signal intensity) initial enhancement (IE) behaviour in both patients with (n = 37, group 1) and patients without (n = 26, group 2) current digital ulcers and/or pitting scars (DU/PS) at the time of the examination. However 67.6% of group 1 (compare *lower left* image of Fig. 2, e.g. digit III left) and 61.5% of group 2 showed a missing strong signal in region 0 or region 1 (see Fig. 4 for regions) during IE in at least one finger.

A total of 57.7% of group 1’s fingers showed a strong initial ICG accumulation in the fingertip (region 0) and 1.9% slightly below the fingertip (region 1, see Fig. 2 *upper left* image), in contrast to 80.9% and 3.0% of group 2’s fingers respectively (*p* < 0.0001). Fingers with a strong IE more proximal than that, were more likely to belong to patients with current digital ulcers and/or pitting scars (group 1): between 63.5% and 100% of fingers with a strong IE in the areas between region 2 (near distal interphalangeal joint, DIP; 74.5%) and region 8 (wrist; 100%) belonged to patients of group 1 (for regions, compare Fig. 4).

Defining a strong IE in region 2 as a cutoff for pathologic enhancement behaviour (strong initial ICG accumulation in proximal areas of the finger/hand instead of the fingertips as seen in most healthy subjects), we calculated 40% sensitivity and 84% specificity for the detection of patients with present DU/PS. The positive predictive value was 73.5%.

### Individual finger analysis in SSc patients shows link between pathologic FOI findings and presence of digital ulcers and/or pitting scars in the same finger

**Fig. 6:**
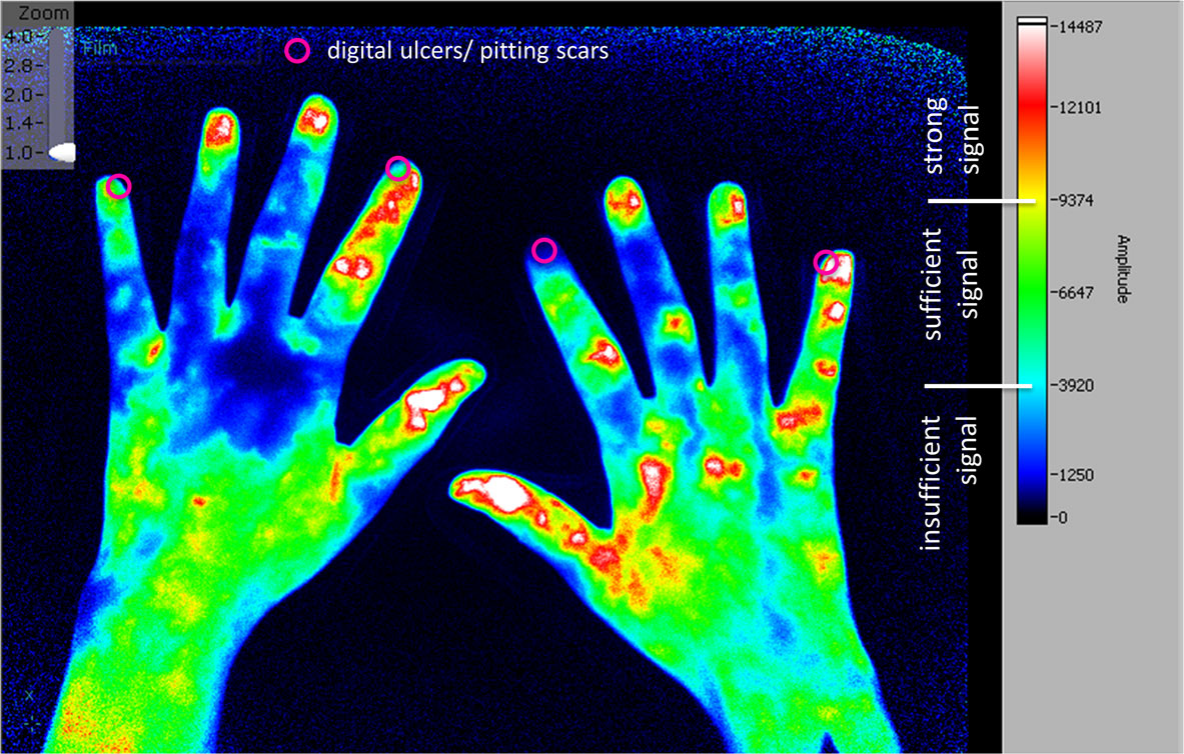
Location of digital ulcers and pitting scars in patients with pathologic FOI. Exemplary depiction of the location of current digital ulcer and/or pitting scars (DU/PS) projected onto a fluorescence optical imaging frame 62 seconds after application of indocyanine green (ICG). The initial enhancement (IE) has already taken place in all fingers with the first strong signal proximal to region 0 in digit II of the right hand

Individual finger analysis showed a strong IE in the fingertips in 78.5% (n = 314 of 400 fingers) of patients with lcSSc (compare FOI staining Fig. 3) and in 43.3% (n = 97 of 224 fingers; six not assessable; *p* < 0.0001) of the patients with dcSSc (see Fig. 2 for FOI staining; compare overview of IE regions in Fig. 4b and c). In SSc patients, a missing initial ICG accumulation in the fingertip significantly more often coincided with the presence of at least one digital ulcer and/or pitting scar in the corresponding finger (see Table 2, exemplary depiction in Fig. 6): 60.9% of fingers with current DU/PS, 26.1% of fingers without these complications showed a strong initial ICG accumulation in more proximal areas (*p* < 0.0001).

**Table 2:** Comparison of fingers with pathologic FOI findings with the presence of current digital ulcers and/or pitting scars (DU/PS) in the same finger (SSc patients)

	Initial enhancement (IE)		Maximum distal distribution (MDD)	
	n (total)^a^	Strong signal proximal of fingertip, n (%)	n (total)^a^	Missing or insufficient signal in fingertip during entire examination, n (%)
Fingers with current DU/PS	138	84 (60.9%)	143	17 (11.9%)
Fingers without current DU/PS	476	124 (26.1%)	477	9 (1.9%)
*p* value (Fisher’s exact *t* test)		*p* < 0.0001		*p* < 0.0001

^a^Note different n (total) for IE and MDD due to six not assessable values for IE

In six fingers of one SSc patient, the initial enhancement could not be determined due to severe sclerodactyly.

After initial enhancement, the maximum distal distribution (MDD) of ICG was observed. A total of 594 fingers showed a sufficient ICG distribution (i.e. ≥27% of maximum signal intensity) in the fingertip in the course of the examination. A complete disruption of ICG accumulation (i.e. <27% of maximum signal intensity in the fingertip at all times) was found in 26 fingers.

In all fingers with current DU/PS, 11.9% showed a pathologic MDD (<27% of maximum signal intensity in the fingertip) compared to only 1.9% in fingers without DU/PS in the corresponding finger (see Table 2). The proportion of 1.9% belonged to four patients especially at risk for the development of future digital ulcers, as they all shared a history of digital ulcers and at least one of the following characteristics: DU in other fingers, late pattern capillaroscopy and diffuse SSc subtype.

### Pathologic FOI findings were associated with other clinical findings

The distribution of ICG differed in patients with SSc from the healthy cohort. Irregular ICG accumulation patterns (“islands”) were present in 50.8% (n = 32 of 63) of SSc patients compared to 21.4% (n = 6 of 28) of the healthy cohort (*p* = 0.0112). Furthermore, 66.7% of SSc (n = 42 of 63 patients) and 25.0% of the healthy cohort (n = 7 of 28) revealed a missing strong signal in at least one fingertip during initial enhancement (*p* = 0.0009). These 42 SSc patients significantly more often reported episodes of Raynaud’s phenomenon in the past week (97.6% vs. 76.2%; *p* = 0.0134). A complete disruption of microcirculation of at least one finger (insufficient MDD) was found in 11 (17.5%) SSc patients, but in none of the healthy subjects.

As seen in Table 3, the modified Rodnan skin score is significantly higher in patients with pathologic IE than in patients with normal IE (*p* = 0.0234). The same is true for the MDD (*p* = 0.0003). Similar results were obtained when focusing on the hands’ skin scores: at least one pathologic finger in IE or MDD was significantly associated with a higher skin score in both hands (IE *p* = 0.0037; MDD *p* < 0.0001). Lastly, similar relations could also be found when analyzing each hand individually: if one finger had a pathologic IE or MDD, the mean skin score of the hand (range 0–6) was higher as well.

**Table 3:** Skin score of SSc patients depending on the results of fluorescence optical imaging

Mean skin score [±SD]	Initial enhancement			Maximum distal distribution		
	Pathologic^a^	Normal	*p* value	Pathologic^a^	Normal	*p* value
Total (range 0–51)	10.2 [±8.2]	6.4 [±7.6]	*p* = 0.0234	17.3 [±8.9]	7.2 [±6.8]	*p* = 0.0003
Hands (range 0–12)	5.4 [±2.9]	3.2 [±2.7]	*p* = 0.0037	8.24 [±2.3]	3.9 [±2.6]	*p* < 0.0001
Left hand (range 0–6)	2.8 [±1.5]	1.6 [±1.3]	*p* = 0.0013	3.9 [±1.1]	2.0 [±1.4]	*p* = 0.0018
Right hand (range 0–6)	2.8 [±1.6]	1.8 [±1.4]	*p* = 0.0163	4.3 [±1.2]	2.0 [±1.4]	*p* = 0.0002

^a^At least one finger with pathologic findings in the respective patient (first two rows) or hand (last two rows)

We also found considerable differences regarding the total score of ACR/EULAR criteria between SSc patients presenting a complete disruption and those with sufficient perfusion. Patients with a pathologic MDD presented a higher mean score (mean: 21.4 points, SD: ±3.6, 95%CI: 18.9–23.8) than those with sufficient ICG accumulation in region 0 (compare Fig. 4) during the course of the examination (mean: 15.7 points, SD: ±3.9, 95%CI: 14.6–16.8; *p* = 0.0005).

### Capillaroscopy and digital ulcers/pitting scars - increasing specificity using FOI

Sixty-one of the 63 SSc patients received a nailfold capillaroscopy examination. Only one patient showed non-SSc-specific alterations in their nailfold. An early pattern was found in 17.5%, an active pattern in 39.7% and a late pattern in 38.1% of SSc patients (compare Fig. 5). In total, 77.1% of all assessable fingers showed a decreased capillary density (<7 capillaries/mm), and 34.1% had one or more megacapillaries (diameter >50 μm). Thirty-eight patients presented megacapillaries in at least one finger, while no megacapillaries could be found in the capillaroscopy of 18 patients. Nevertheless, almost half of these 18 patients (44.4%) had current DU/PS. A total of 63.2% of patients with megacapillaries showed DU/PS.

A higher percentage of SSc patients with pathologic MDD presented a late pattern in the nailfold capillaroscopy (81.8% vs. 30.0%, *p* = 0.0043).

The calculated sensitivity and specificity for nailfold capillaroscopy were 86.1% and 28.0%, respectively, opposing non-SSc and early pattern to active and late pattern in regard to present DU/PS. The positive predictive value amounted to 63.3%.

Combining the capillaroscopy with an additional FOI examination revealed the following: while sensitivity dropped to FOI levels of 40.4%, specificity increased dramatically to 88.4%. The positive predictive value improved and rose to 83.1%.

### Intra- and inter-reader reliability

The intra-reader reliability amounted to 0.786; only 7.6% of the initial assessment differed from the second rating of examiner 1. The inter-reader reliability was 0.834 with 95.7% accordance between the assessments of examiner 1 (second assessment) and examiner 2.

### Safety

In the included cohorts, no side effects to ICG application were observed.

## Discussion

In the present study, fluorescence optical imaging (FOI) was used to investigate microvasculopathy in SSc. As presented above, the SSc group was easily distinguishable from young healthy subjects by their FOI staining (compare Figs. 1, 2, 3 and 4). While the healthy cohort mainly presented a strong initial ICG enhancement in the fingertips, the SSc group often showed strong initial ICG signals in more proximal areas. Furthermore, a complete disruption (i.e. insufficient signal in the fingertips in the course of the examination) of digital perfusion was specifically observed in SSc patients. Pathologic FOI staining was associated with diffuse SSc subtype, late pattern in NC, a higher modified Rodnan skin score and a higher total score of ACR/EULAR classification criteria. Additionally, individual finger analysis revealed associations with present digital ulcers or pitting scars (DU/PS) in the corresponding finger. FOI presented a relatively high specificity regarding its ability to distinguish patients with and without these complications. No significant associations of FOI results and smoking habits, disease duration or BMI were found (data not shown).

While a strong screening method due to its high sensitivity, one of capillaroscopy’s drawbacks is the low specificity regarding capillaroscopy patterns. A combination of capillaroscopy and FOI showed an important specificity increase when comparing patients with and without current DU/PS. Therefore, FOI can be considered as a new, relatively fast and promising tool that could be helpful in the assessment and the exact localization of malperfused areas in patients with systemic sclerosis. So far, FOI has mainly been used in the diagnosis of arthritis such as rheumatoid arthritis and psoriatic arthritis. They focused on areas of increased signal intensity by analysing ICG distribution in order to identify joint inflammation [1–3]. Pfeil et al. made first attempts to examine patients with SSc by FOI to visualise inflamed tissue [7]. This greatly differs from the novel approach taken in the present work as it represents an effort to examine and quantify tissue nutrition and malperfusion instead of inflammation. We focused on the analysis of (low) ICG enhancement to measure the arterial distribution of the colouring agent, which revealed areas with insufficient FOI staining in SSc patients. Associations between FOI enhancement patterns and present signs of ischemia such as digital ulcers and pitting scars support our approach.

There have been a few other emerging imaging methods trying to investigate peripheral blood perfusion in SSc patients [30]: different techniques have shown promising results in recent years, among them laser speckle contrast analysis (LASCA) [14, 31], laser Doppler imaging (LDI), laser speckle contrast imaging (LSCI) and laser Doppler flowmetry (LDF); all of them non-invasive. [30] LASCA, LDI and LDF proved to discriminate SSc patients from those with primary Raynaud’s phenomenon and/or healthy subjects. [32–34] Low blood flow detected by LASCA and LDF was associated with the late pattern in NC [33, 35] and improved under vasoactive therapies [14, 33]. There have also been first results showing that colour Doppler ultrasonography of the arteries of the fingers and hands is linked to the presence of digital ulcers/pitting scars [36]. Other new techniques that are being investigated momentarily are thermography and optical Doppler tomography and spectroscopy [30].

Similar to LASCA and LDF, we showed that FOI was able to discriminate between SSc patients and healthy controls and that its findings were associated with a late pattern capillaroscopy. Pfeil et al. [7] also demonstrated a change in FOI pattern under vasoactive treatment, which should be investigated for this study’s protocol in the future. One of FOI’s limitations might be the intravenous application of ICG, especially in comparison to those non-invasive methods to measure blood perfusion and capillaroscopy. While not reaching sensitivity and specificity levels of the CSURI (capillaroscopic skin ulcer risk index), analysis of FOI findings are not limited to the presence of megacapillaries and therefore applicable to almost all SSc patients (i.e. patients with early pattern capillaroscopy) [16, 17, 25]. Another aspect, which needs to be considered when interpreting FOI results, is the proportional character of the results. As the total ICG signal varies depending on factors like the current cardiovascular state of the subject, the speed of the injection, or the effective concentration of ICG in the blood, the signal is depicted as a proportion of the strongest signal in the examined area. As a consequence, FOI depicts microvascular disturbances in the hand, not the absolute blood volume reaching the hand.

FOI was conducted in a mean time of 15 minutes – including preparation and analysis. Also, the examination itself can be run by a trained medical assistant and does not require the presence of a medical physician until the point of analysis. This time-saving side of FOI could prove a valuable asset in clinical daily practice where most patients already present with intravenous catheters. Furthermore, our study revealed a considerable level of reliability for this new method.

While the comparison of SSc patients and healthy subjects in this report is of cross-sectional design, SSc patients will be further investigated as part of a longitudinal study with the objective to identify the predictive value of FOI and other diagnostic methods regarding the development of future digital ulcers.

Study limitations include the relatively low number of participants as well as the age differences between the healthy cohort and the SSc group. Due to the centre’s speciality, patients with severe systemic sclerosis were more frequent in our cohort than in the SSc population (i.e. higher incidence of DU/PS). We also observed difficulties in the analysis of FOI staining in patients with severe sclerodactyly. Even though our primary objective was the assessment of microcirculation, we observed an overlap of seemingly inflamed joints (as seen in *PrimaVistaMode*, the composite image of the examination) and the region of initial ICG enhancement in small share of patients (data not shown). Here, an interference of inflammation and initial enhancement cannot completely be ruled out. Additionally, Pfeil et al. [7] described areas of ICG enhancement, which were interpreted as soft tissue inflammation. While we focused on areas of diminished perfusion, the potential interactions of joint as well as soft tissue inflammation and circulation disturbances should be investigated in more detail in future studies.

While we examined three patients with primary Raynaud’s (unpublished data) who mostly showed enhancement patterns similar to the healthy cohort, a more extensive investigation into FOI in primary Raynaud’s patients as well as a comparison to patients with secondary Raynaud’s is needed.

## Conclusions

Further investigations into FOI’s ability to detect abnormal microcirculation are suggested to prove FOI validation data in SSc patients. A software for automatically generated disease activity values (DACT) has been developed for patients with inflamed joints, but not yet for areas of malperfusion in the hands.

The present work and subsequent results represent first attempts at a broader detection of vasculopathy in SSc using the FOI method. FOI could be a novel tool for the assessment of symptomatic vasculopathy and could provide additional assistance in the risk stratification of digital ulcers.

## Abbreviations

BMBF: Bundesministerium für Bildung und Forschung (German Federal Ministry for Education and Research)
CAP: VideoCAPillaroscopy (multicentre study)
CCD: Charge-coupled detector
CSURI: Capillaroscopic skin ulcer risk index
DACT-FOI: Digital activity in fluorescence optical imaging (software tool)
DcSSc: Diffuse cutaneous systemic sclerosis
DU: Digital ulcers
FOI: Fluorescence optical imaging
GFR: Glomerular filtration rate
ICG: Indocyanine green (fluorophore)
IE: Initial enhancement
LASCA: Laser speckle contrast analysis
L(c)SSc: Limited (cutaneous) systemic sclerosis
LDF: Laser Doppler flowmetry
LDI: Laser Doppler imaging
LSCI: Laser speckle contrast imaging
MDD: Maximum distal distribution
MRI: Magnetic resonance imaging
NC: Nailfold capillaroscopy
PPG: Photoplethysmography
PS: Pitting scars
RP: Raynaud’s phenomenon
SSc: Systemic sclerosis
US: Ultrasound

## References

[1] Werner SG, Langer HE, Schott P (2013). Indocyanine green-enhanced fluorescence optical imaging in patients with early and very early arthritis: a comparative study with magnetic resonance imaging. Arthritis Rheum.

[2] Glimm AM, Werner SG, Burmester GR, et al. Analysis of distribution and severity of inflammation in patients with osteoarthitis compared to rheumatoid arthritis by ICG-enhanced fluorescence optical imaging and musculoskeletal ultrasound: a pilot study. Ann Rheum Dis. 2016;75(3):566–70. doi:10.1136/annrheumdis-2015-207345. 10.1136/annrheumdis-2015-207345 PMC4789689 26311723

[3] Fischer T, Ebert B, Voigt J (2010). Detection of rheumatoid arthritis using non-specific contrast enhanced fluorescence imaging. Acad Radiol.

[4] Mothes H, Dönicke T, Friedel R (2004). Indocyanine-green fluorescence video angiography used clinically to evaluate tissue perfusion in microsurgery. J Trauma.

[5] Benya R, Quintana J, Brundage B (1989). Adverse reactions to indocyanine green: a case report and a review of the literature. Cathet Cardiovasc Diagn.

[6] Holzbach T, Taskov C, Henke J (2005). Evaluation of perfusion in skin flaps by laser-induced indocyanine green fluorescence. Handchir Mirkrochir Plastchir.

[7] Pfeil A, Drummer KF, Boettcher J (2015). The application of fluorescence optical imaging in systemic sclerosis. Biomed Res Int.

[8] Botzoris V, Drosos AA (2011). Review: Management of Raynaud’s phenomenon and digital ulcers in systemic sclerosis. Joint Bone Spine.

[9] Sunderkötter C, Herrgott I, Brückner C (2009). Comparison of patients with and without digital ulcers in systemic sclerosis: detection of possible risk factors. Br J Dermatol.

[10] Silva I, Almeida J, Vasconcelos C (2015). Review: A PRISMA-driven systematic review for predictive risk factors of digital ulcers in systemic sclerosis patients. Autoimmun Rev.

[11] Caramaschi P, Canestrini S, Martinelli N (2007). Scleroderma patients nailfold videocapillaroscopic patterns are associated with disease subset and disease severity. Rheumatology (Oxford).

[12] Silva I, Teixeira A, Oliveira J (2015). Endothelial dysfunction and nailfold videocapillaroscopy pattern as predictors of digital ulcers in systemic sclerosis: a cohort study and review of the literature. Clin Rev Allergy Immunol.

[13] McKay N, Griffiths B, Di Maria C (2014). Novel photoplethysmography cardiovascular assessments in patients with Raynaud’s phenomenon and systemic sclerosis: a pilot study. Curr Opin Rheumatol.

[14] Ruaro B, Sulli A, Smith V (2015). Short-term follow-up of digital ulcers by laser speckle contrast analysis in systemic sclerosis patients. Microvasc Res.

[15] Meijs J, Voskuyl AE, Bloemsaat-Minekus JP (2015). Blood-flow in the hands of a predefined homogeneous systemic sclerosis population: the presence of digital ulcers and the improvement with bosentan. Rheumatology (Oxford).

[16] Sebastiani M, Manfredi A, Colaci M (2009). Capillaroscopic Skin Ulcer Risk Index: a new prognostic tool for digital skin ulcer development in systemic sclerosis patients. Arthritis Rheum.

[17] Sebastiani M, Manfredi A, Vukatana G (2012). Predictive role of capillaroscopic skin ulcer risk index in systemic sclerosis: a multicentre validation study. Ann Rheum Dis.

[18] Smith V, De Keyser F, Pizzorni C (2011). Nailfold capillaroscopy for day-to-day clinical use: construction of a simple scoring modality as a clinical prognostic index for digital trophic lesions. Ann Rheum Dis.

[19] Cutolo M, Herrick A, Distler O, et al. Nailfold videocapillaroscopy and clinical characteristics to predict digital ulcer risk in systemic sclerosis: a multicenter, prospective cohort study. Arthritis Rheumatol. 2016;68(10):2527–39. doi:10.1002/art.39718. 10.1002/art.39718 PMC5129545 27111549

[20] Smith V, Decuman S, Sulli A (2012). Do worsening scleroderma capillaroscopic patterns predict future severe organ involvement? A pilot study. Ann Rheum Dis.

[21] Cutolo M, Sulli A, Pizzorni C, Accardo S (2000). Nailfold videocapillaroscopy assessment of microvascular damage in systemic sclerosis. J Rheumatol.

[22] Smith V, Thevissen K, Trombetta AC (2016). Nailfold capillaroscopy and clinical applications in systemic sclerosis. Microcirculation.

[23] Smith V, Riccieri V, Pizzorni C (2013). Nailfold capillaroscopy for prediction of novel future severe organ involvement in systemic sclerosis. J Rheumatol.

[24] Ingegnoli F, Ardoini I, Boracchi P, Cutolo M (2013). EUSTAR co-authors. Nailfold capillaroscopy in systemic sclerosis: DATA from the EULAR scleroderma trials and research (EUSTAR) database. Microvasc Res.

[25] Medsger TA, Silman AJ, Steen VD (1999). A disease severity scale for systemic sclerosis: development and testing. J Rheumatol.

[26] van den Hoogen F, Khanna D, Fransen J (2013). 2013 classification criteria for systemic sclerosis: an American College of Rheumatology/European League against Rheumatism collaborative initiative. Ann Rheum Dis.

[27] LeRoy EC, Medsger TA (2001). Criteria for the classification of early systemic sclerosis. J Rheumatol.

[28] Cherrick GR, Stein SW, Leevy CM, Davidson CS (1960). Indocyanine green: observations on its physical properties, plasma decay, and hepatic extraction. J Clin Invest.

[29] Granzow N, Schoenberger S, Ohrndorf S (2011). Evaluation of a novel fluorescence optical imaging technology in patients with connective tissue diseases and arthralgia compared to musculoskeletal ultrasonography. Ann Rheum Dis.

[30] Pizzorni C, Sulli A, Smith V (2016). Capillaroscopy in 2016: new perspectives in systemic sclerosis. Acta Reumatol Port.

[31] Lambrecht V, Cutolo M, De Keyser F (2016). Reliability of the quantitative assessment of peripheral blood perfusion by laser speckle contrast analysis in systemic sclerosis cohort. Ann Rheum Dis.

[32] Murray AK, Moore TL, Manning JB (2009). Noninvasive imaging techniques in the assessment of scleroderma spectrum disorders. Arthritis Rheum.

[33] Cutolo M, Ferrone C, Pizzorni C (2010). Peripheral blood perfusion correlates with microvascular abnormalities in systemic sclerosis: a laser-Doppler and nailfold videocapillaroscopy study. J Rheumatol.

[34] Sulli A, Ruaro B, Cutolo M (2014). Evaluation of blood perfusion by laser speckle contrast analysis in different areas of hands and face in patients with systemic sclerosis. Ann Rheum Dis.

[35] Ruaro B, Sulli A, Alessandri E (2014). Laser speckle contrast analysis: a new method to evaluate peripheral blood perfusion in systemic sclerosis patients. Ann Rheum Dis.

[36] Lüders S, Friedrich S, Ohrndorf S et al. Detection of severe vasculopathy in systemic sclerosis by colour Doppler sonography is associated with digital ulcers. Rheumatology (Oxford). 2017. doi: 10.1093/rheumatology/kex045. 10.1093/rheumatology/kex045 28340234

